# Susceptibility of Field and Laboratory Bt-Susceptible and Resistant Strains of *Helicoverpa zea* (Boddie) to HearNPV

**DOI:** 10.3390/plants13040529

**Published:** 2024-02-15

**Authors:** Wilfrid Calvin, Fei Yang, Haley Kennedy, Paula G. Marçon, David L. Kerns

**Affiliations:** 1 Department of Entomology, Texas A&M University, 2475 TAMU, College Station, TX 77843, USA; calvi095@umn.edu (W.C.); haleykennedy7@tamu.edu (H.K.); 2 Department of Entomology, University of Minnesota, Saint Paul, MN 55108, USA; 3 AgBiTech, Fort Worth, TX 76155, USA; pmarcon@agbitech.com

**Keywords:** *Helicoverpa zea*, HearNPV, IPM, resistance management, biological insecticide

## Abstract

During 2021 and 2022, eight field-collected and five laboratory *Helicoverpa zea* strains with varying susceptibility to different Bt proteins were evaluated for their responses against HearNPV using diet-overlay bioassays. The five laboratory strains included SS (susceptible to all Bt proteins), CRY-RR (resistant to Cry1 and Cry2), VIP-RR-70 (resistant to Vip3Aa), VIP-RR-15 (resistant to Vip3Aa), and TRE-RR (resistant to Cry1, Cry2, and Vip3Aa). Our findings showed that the susceptibility of TRE-RR, VIP-RR-70, and VIP-RR-15 strains to HearNPV was similar to that of the SS strain. However, the field and Cry-RR strains were more resistant to HearNPV compared to the SS strain. Because most feral *H. zea* strains in the southern U.S. have developed practical resistance to Cry Bt proteins but remain susceptible to Vip3Aa, the results suggest that the reduced susceptibility to HearNPV in *H. zea* may be associated with the resistance to Cry Bt proteins but not with the resistance to Vip3Aa. Correlation analysis confirmed that there was a significant positive relationship between Cry resistance and HearNPV resistance, but not between the Vip3Aa resistance and HearNPV resistance in *H. zea.* Our findings provide valuable insights into the relationship between susceptibility to HearNPV and resistance to Bt proteins in *H. zea*.

## 1. Introduction

The cotton bollworm (*Helicoverpa zea* Boddie) is a polyphagous insect pest that can attack many agricultural crops, including corn (*Zea mays* L.), sorghum (*Sorghum bicolor* (L.) Moench), cotton (*Gossypium* spp.), soybeans (*Glycine max* (L.) Merr.), tomatoes (*Solanum lycopersicum* L.), hemp (*Cannabis sativa* L.), cabbage (*Brassica oleracea* L.), cowpeas (*Vigna unguiculata* (L.) Walp), cucumber (*Cucumis sativus* L.), and eggplant (*Solanum melongena* L.) [1,2,3,4,5]. *Helicoverpa zea* is primarily controlled using conventional insecticides and Bt plant-incorporated protectants [6,7]. However, this pest is notorious for developing resistance to insecticides and has developed resistance to various classes of insecticides, such as organochlorines (IRAC MoA group 2A), organophosphates (IRAC MoA group 1B), and pyrethroids (IRAC MoA group 3A). In recent years, *H. zea* has also developed resistance to Cry1 and Cry2 Bt proteins (IRAC MoA group 11A) [6,8,9,10,11,12]. The development of resistance to insecticides and Bt proteins by *H. zea* poses a significant challenge for pest management.

Effective management of *H. zea* populations in the field can be achieved through the implementation of integrated pest management (IPM) approaches by mitigating the already established resistance and delaying the onset of resistance to new insecticide classes [13,14]. *Helicoverpa armigera* nucleopolyhedrovirus (HearNPV) is a type of entomopathogenic virus belonging to the family Bacculoviridae [15]. It selectively targets lepidopteran insects in the subfamily Heliothinae, including *H. zea* [16]. HearNPV-based insecticides are host-specific and selective with a novel mode of action (IRAC MoA group 31) [15,17]. Based on these characteristics, incorporating HearNPV into an IPM program targeting *H. zea* may be a highly effective strategy. In fact, HearNPV has already been adopted to a limited extent for managing *H. zea* in soybeans, grain sorghum, and hemp in the U.S., and researchers have investigated its potential to manage *H. zea* in cotton as well [18,19,20,21].

HearNPV can be applied as a foliar spray like conventional insecticides and is activated upon larval ingestion. The virus infects the host with two phases, namely primary and secondary infection. During the primary phase, the larvae ingest the virus occlusion bodies (OBs) that protect the virus from environmental conditions. These OBs then travel through the digestive tract of the larvae and reach the midgut. Once in the insect midgut, the alkaline content of the midgut activates the virus by degrading the occlusion bodies, causing the release of occlusion-derived virions. These virions then cross the peritrophic membrane, infect the midgut cells, and replicate. In the second phase of the infection, the replicated virus egresses as a budded virus and spreads throughout the host’s muscle, fat body, hemocytes, and tracheal cells, causing the host to liquefy [15,22,23]. When the host larva dies and liquefies, millions of viral particles are released into the environment, which can result in horizontal and abiotic transmissions [24].

Resistance to baculovirus in insects has been reported in several studies. For instance, *H. zea* and the African cotton leafworm (*Spodoptera littoralis* Boisduval) have shown resistance to *Autographa californica* multiple nucleopolyhedrovirus (*Ac*MNPV) infections. These insects developed resistance to the virus by melanizing and encapsulating the tracheal epidermis, a mechanism that prevents secondary infection from occurring [25]. In Germany, there have been several reports of field resistance of the codling moth (*Cydia pomonella* L.) to *Cydia pomonella* Granulovirus (*Cp*GV). In resistant *C. pomonella*, the virus can cross the peritrophic membrane, but the larva provokes a systemic blockade that prevents the viral DNA replication within the midgut cells [26,27]. Additionally, *Baculovirus heliothis* resistance has also been reported in *Heliothis subflexa* (Guenée), but the mechanism of this resistance is unknown [28]. 

Developing new insecticides for pest management is a challenging and costly process that is also subject to strict regulatory requirements [29]. As HearNPV-based insecticides become more prominent Lepidoptera management tools, it is imperative to establish proactive Insecticide Resistance Management (IRM) programs for the long-term use of HearNPV. Understanding the baseline susceptibility of insect pests to insecticides is essential for a resistance monitoring program [30,31]. Therefore, it is critically important to determine the baseline susceptibility and cross-resistance of *H. zea* to HearNPV before the widespread adoption of this new insecticide mode of action group. The objective of this study is to understand the efficacy and baseline susceptibility of several field and laboratory Bt-susceptible and resistant strains of *H. zea* to HearNPV.

## 2. Results

### 2.1. Dose-Response Bioassays in 2021

**Figure 1:**
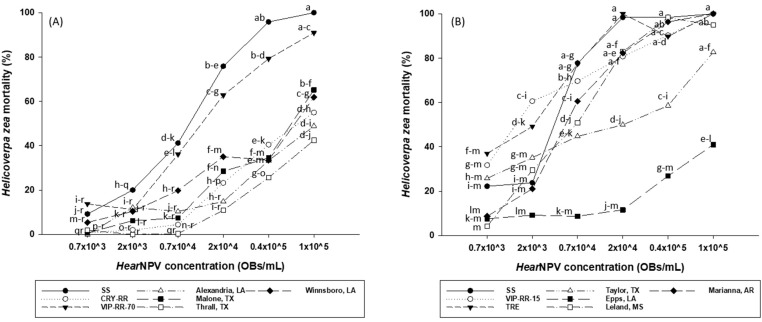
Mean percent mortality among *Helicoverpa zea* strains as affected by HearNPV concentrations in 2021 (**A**) and 2022 (**B**). Means with the same letter are not statistically different (*p* > 0.05). OBs/mL = occlusion bodies per milliliter.

In 2021, the effects of insect strain and HearNPV concentration on larval mortality were significant (F = 52.47; df = 6, 131.3; *p <* 0.0001 for insect strain and F = 137.04; df = 5, 131; *p <* 0.0001 for HearNPV concentration). The interactions between insect strain and HearNPV concentration were also significant (F = 2.15; df = 30, 131; *p =* 0.0017). SS *H. zea* was susceptible to HearNPV with an estimated LC_50_ value of 6.5 × 10^3^ OBs/mL (Table 1) and 100.0% mortality at the highest tested concentration of 1 × 10^5^ OBs/mL (Figure 1A). VIP-RR-70 strain, which is susceptible to Cry1Ac and Cry2Ab2 (Table 2), was also susceptible to HearNPV with an estimated LC_50_ value of 10.5 × 10^3^ OBs/mL and 91.0% mortality at the HearNPV concentration of 1 × 10^5^ Obs/mL (Table 1 and Figure 1A). Based on the overlapping of 95% confidence intervals of LC_50_s, there were no significant differences in susceptibility against HearNPV between the SS and VIP-RR-70 strains. Relative to SS, the resistance ratio for VIP-RR-70 was 1.6-fold against HearNPV (Table 1). In contrast, the Cry-RR and all field strains that are highly resistant to Cry proteins (Table 2) exhibited significant levels of resistance against HearNPV relative to SS, based on the non-overlapping of 95% CLs (Table 1). In addition, the mortality of Cry-RR and field strains of *H. zea* were significantly (*p* < 0.05) lower than that of SS at the high HearNPV concentrations of 2 × 10^4^–1 × 10^5^ OBs/mL (Figure 1A). Relative to SS, the resistance ratios for Cry-RR and field strains ranged from 9.5- to 22.7-fold (Table 1).

### 2.2. Dose-Response Bioassays in 2022

In 2022, the effects of insect strain, HearNPV concentration, and the interactions between insect strain and HearNPV concentration on larval mortality were all significant (F = 48.71; df = 6, 113.4; *p <* 0.0001 for insect strain; F = 122.89; df = 5, 113; *p <* 0.0001 for HearNPV concentration; F = 4.16; df = 30, 113; *p <* 0.0001 for the interactions). Similarly, SS showed high susceptibility against HearNPV, with an estimated LC_50_ value of 2.8 × 10^3^ OBs/mL (Table 1) and 100.0% mortality at the highest HearNPV concentration (Figure 1B). The VIP-RR-15 strain of *H. zea*, which is resistant to Vip3Aa Bt proteins but susceptible to Cry1Ac and Cry2Ab2 bt proteins, was also susceptible to HearNPV with an estimated LC_50_ value of 1.8 × 10^3^ OBs/mL and 100.0% mortality at the HearNPV concentration of 1 × 10^5^ OBs/mL (Table 1 and Figure 1B). Based on the overlapping of 95% confidence intervals of LC_50_s, there were no significant differences in susceptibility against HearNPV between the SS and VIP-RR-15 strains. Compared to SS, the resistance ratio for VIP-RR-15 was 0.7-fold against HearNPV (Table 1). By contrast, all field strains that are highly resistant to Cry proteins (Table 2), exhibited significant levels of resistance against HearNPV compared to SS based on the non-overlapping of 95% CLs (Table 1). Relative to SS, the resistance ratios for these field strains ranged from 1.8-to 175.0-fold (Table 1). The TRE-RR strain, which is resistant to Cry1Ac, Cry2Ab2, and Vip3Aa Bt proteins, was susceptible to HearNPV with an estimated LC_50_ value of 1.6 × 10^3^ OBs/mL, resulting in a resistance ratio of 0.7-fold against HearNPV relative to SS (Table 1). 

### 2.3. Correlation Analysis between HearNPV and Bt Resistance

The resistance ratio data for HearNPV and Cry1Ac, Cry2Ab2, or Vip3Aa Bt proteins in Table 1 and Table 2 were used in the correlation analysis to explore the potential relationship between HearNPV and Bt resistance in *H. zea*. Due to a statistical outlier (resistance ratio for Epps-LA strain) in the pair of Cry1Ac and HearNPV, two separate correlation analyses were conducted for exploring the relationship between HearNPV and Cry1Ac resistance, one using the complete data set and another with the outlier removed. 

When analyzing the complete data set for HearNPV and Cry1Ac resistance in *H. zea*, there was no significant correlation observed between Cry1Ac and HearNPV resistance (r = 0.02, *p* = 0.9438). However, after removing the outlier, a highly significant positive correlation was found between HearNPV and Cry1Ac resistance (r = 0.81, *p* = 0.0015). Regarding Cry2Ab2 resistance, a significant positive correlation was detected between Cry2Ab2 and HearNPV resistance (r = 0.67, *p* = 0.0089). On the other hand, we did not observe any significant correlation between the Vip3Aa and HearNPV resistance ratios (r = −0.17, *p* = 0.5566). 

## 3. Discussion

The widespread development of practical resistance to Cry Bt proteins in *H. zea* in the U.S. highlights the urgent need for alternative pest management strategies and novel insecticides with different modes of action [36]. To address this challenge, incorporating baculoviruses like HearNPV into Integrated Pest Management (IPM) and Insect Resistance Management (IRM) programs for *H. zea* is a promising approach. HearNPV offers high efficacy, specificity, and selectivity, as demonstrated by previous studies that showed its effectiveness and low variation in susceptibility across field populations and the susceptible strain of the closely related species, old world bollworm (*Helicoverpa armigera* Hübner) [17]. Despite its potential, the adoption of HearNPV for managing *H. zea* in various agricultural crops in the U.S. has been limited [18,19,20,37,38]. Expanding the use of HearNPV could significantly contribute to mitigating insecticide and Bt resistance in *H. zea*. In this study, we conducted the first investigation to determine the baseline susceptibility to HearNPV of multiple field and laboratory strains of *H. zea*, which exhibited varying susceptibility levels to different Bt proteins. Furthermore, we aimed to explore any potential relationship between HearNPV and Bt resistance in *H. zea*.

Based on the findings of our study, *H. zea* strains displaying resistance to Cry1Ac and/or Cry2Ab2 Bt proteins exhibited significant reduced susceptibility to HearNPV compared to the genetically similar SS strain based on the non-overlapping of 95% CLs. Furthermore, correlation analysis based on resistance ratios revealed positive associations between HearNPV resistance and Cry1Ac and/or Cry2Ab2 in *H. zea*. In contrast, *H. zea* strains resistant to Vip3Aa Bt protein exhibited a high level of susceptibility to HearNPV similar to the SS strain, and correlation analysis showed no significant correlation between the Vip3Aa and HearNPV resistance in *H. zea*. However, the TRE-RR strain, which is resistant to Cry1, Cry2, and Vip3Aa Bt proteins, remained highly susceptible to HearNPV. Although the underlying reason for this high susceptibility is unclear, it provides further evidence of the value of HearNPV as a new resistance management tool. In a similar study, Cry2Ab/Vip3A-resistant strains of *H. armigera* and the Australian bollworm (*Helicoverpa punctigera* Wallengren) exhibited LC_50_ values of HearNPV comparable to their respective reference-susceptible strains [39]. 

Due to the failures of Cry1 and Cry2 Bt proteins and the growing use of Vip3Aa expressing corn and cotton, there is now increased selection pressure for Vip3Aa resistance in *H. zea* in the U.S. Therefore, it is urgent to integrate alternative strategies into the IPM and IRM program to preserve the durability of the Vip3Aa protein in managing *H. zea.* In this current study, we found that *H. zea* strains susceptible to Cry Bt proteins but resistant to Vip3Aa Bt proteins, and resistant to both Cry and Vip3Aa Bt proteins, remained susceptible to HearNPV. These results suggest that the HearNPV virus has potential in preventing and delaying the evolution of resistance to Vip3Aa in *H. zea*. Due to the rise of resistance to Bt crops in *Helicoverpa* spp., biological control agents are becoming more popular. The renewed interest in alternative biological control agents is also a result of public demand as awareness grows of the potentially detrimental impacts pesticides have on beneficial insects, human health, and the environment [40,41,42]. When applied in conjunction with a holistic Integrated Pest Management (IPM) strategy, NPV-based insecticides provide an effective and environmentally friendly alternative to chemical insecticides [43,44]. Moreover, studies have previously reported the effectiveness of NPV-based insecticides against insecticide-resistant insect pests, including those resistant to Bt proteins [17,39,45,46]. 

Nevertheless, it is important to acknowledge that the risk for HearNPV resistance development exists, particularly if this new mode of action group is used extensively without the proper implementation of Insect Resistance Management (IRM) programs. Therefore, it is of critical importance to develop sustainable and proactive strategies to delay resistance and promote integrated pest management approaches for using HearNPV in *H. zea*. Several strategies can be implemented to mitigate the development of HearNPV resistance in *H. zea.* These approaches include a regular Insect Resistance Monitoring program and the adoption of diverse control measures including crop and insecticide rotation, utilization of biological control agents such as beneficial arthropods (predators and parasitoids), mechanical control, sanitation, and tank-mixes of HearNPV with one or more insecticides that have a distinct mode of action [47,48]. Due to its unique mode of action, HearNPV can effectively contribute to managing the insecticide resistance commonly encountered in *H. zea* control [17]. Additionally, HearNPV as a selective insecticide has no impact on natural enemies and other beneficial arthropods [16,49]. Consequently, the adoption of HearNPV helps preserve beneficial organisms, maximizes biological control, and mitigates the risk of insecticide-induced secondary pest outbreaks [15,17,49]. In conclusion, by characterizing the susceptibility of different *H. zea* strains to HearNPV and investigating its correlation with Bt resistance, our study contributes to understanding the potential of HearNPV as an effective tool in managing *H. zea*. The varying levels of susceptibility to HearNPV among different strains of *H. zea* and its correlations with specific Bt resistance traits warrant further investigation to understand the underlying mechanisms. These findings have practical implications for developing sustainable strategies to effectively combat insecticide resistance and promote integrated pest management approaches for this pest. 

## 4. Materials and Methods

### 4.1. Laboratory Insect Strains 

A susceptible strain (SS) was obtained from Benzon Research Inc. (Carlisle, PA, USA) and has been previously shown to be susceptible to Cry1Ac, Cry2Ab2, Cry1F, and Vip3Aa Bt proteins [50,51]. A CRY-RR strain was established using an F_2_ screen method with populations collected in Snook, Texas in 2018 [50]. This strain is resistant to Cry1Ac, Cry1F, and Cry2Ab2 Bt proteins, but susceptible to the Vip3Aa Bt protein [32]. A VIP-RR-70 strain was isolated through F_2_ screening with moths light-trapped in Snook, Texas in 2019 [50]. A VIP-RR-15 strain was isolated through F_2_ screening with larvae collected in Stoneville, Mississippi in 2020 [52]. Both VIP-RR-70 and VIP-RR-15 strains are highly resistant to the Vip3Aa Bt protein but are susceptible to Cry1Ac, Cry1F, and Cry2Ab2 Bt proteins [50,52]. A TRE-RR strain was derived through the F_2_ method by crossing CRY-RR and VIP-RR-70 strains. The TRE-RR strain has been documented to be resistant to Cry1Ac, Cry2Ab2, and Vip3Aa Bt proteins [33]. Prior to this study, all the original resistant strains of *H. zea* had been backcrossed with SS and reselected for resistance to produce resistant strains that were genetically similar to the SS strain. 

### 4.2. Field-Collected Insect Strains

In 2021, field strains of *H. zea* were collected from Intrasect (Cry1Ab + Cry1F), DoublePro (Cry1A.105 + Cry2Ab2), and Trecepta (Cry1A.105 + Cry2Ab2 + Vip3Aa) corn (Table 2). Laboratory bioassays revealed that the Thrall-TX and Winnsboro-LA strains were resistant to both Cry1Ac and Cry2Ab2, but susceptible to Vip3Aa (Table 2). The Malone-TX strain was resistant to Cry1Ac but was susceptible to Cry2Ab2 and Vip3Aa. The Alexandria-LA strain was not tested for resistance to Cry1Ac but was susceptible to Cry2Ab2 and Vip3Aa. In 2022, the field-collected populations were obtained from non-Bt corn and crimson clover (Table 2). The Epps-LA, Leland-LA, and Taylor-TX strains were all resistant to both Cry1Ac and Cry2Ab2, but susceptible to Vip3Aa, while the Mariana-AR strain was resistant to Cry1Ac, but susceptible to Cry2Ab2 and Vip3Aa (Table 2).

### 4.3. Preliminary Tests for Determining HearNPV Concentrations 

We used Heligen (AgBiTech, Fort Worth, TX, USA), a commercial insecticide containing occlusion bodies (OBs) of the nucleopolyhedrovirus of *Helicoverpa* spp. at a concentration of 7.5 × 10^9^ OBs/mL, as the HearNPV inoculum in this study. To determine the appropriate HearNPV concentrations that could cause mortality in *H. zea* ranging from 0 to 100%, we conducted diet-overlay bioassays using a series of HearNPV concentrations, including 1 × 10, 2 × 10, 3 × 10, 1 × 10^2^, 2 × 10^2^, 3 × 10^2^, 1 × 10^3^, 2 × 10^3^, 3 × 10^3^, 1 × 10^4^, 2 × 10^4^, 3 × 10^4^, 1 × 10^5^, 2 × 10^5^, and 3 × 10^5^ OBs/mL, along with a non-treated control. Based on the preliminary bioassay results, we selected concentrations of 7 × 10^2^, 2 × 10^3^, 7 × 10^3^, 2 × 10^4^, 4 × 10^4^, and 1 × 10^5^ OBs/mL for conducting the full range dose-response bioassays discussed below. 

### 4.4. Dose-Response Bioassays

The aforementioned six concentrations along with a non-treated control were used to determine the susceptibility of *H. zea* to HearNPV in the diet-overlay bioassays. To facilitate visualization of the solution on the diet, a red food dye (McCormick Culinary, Hunt Valley, MD, USA) at a concentration of 1 µL/mL was added to the treatment solutions. In 2021, we performed bioassays using 29 mL solo condiment cups (Dart Container Corporation, Mason, MI, USA), with each cup filled with 6 mL of liquid diet (Southland Product, Inc., Lake Village, AR, USA). After the diet was cooled, we overlaid 100 µL of HearNPV treatment solution onto the surface of the diet. We then used a fine paintbrush to infest each cup with a second instar *H. zea* larva and covered the cups with corresponding lids. Each bioassay was replicated 3 to 5 times with 14 to 30 larvae per replication. In 2022, we switched to using 128-well bioassay trays (C-D International, Pitman, NJ, USA), with each well containing 1 mL of liquid diet. We overlaid each well with 50 µL of HearNPV treatment solutions and infested it with a second instar *H. zea* larva. After infestation, the bioassay trays were sealed with air-vented covers (C-D International, Pitman, NJ, USA), as described in previous studies [32,53]. Each bioassay was repeated 3 to 5 times with 12 to 16 larvae per replication. Both cups and bioassay trays along with infested insects were kept in a climate-controlled room at 25 °C and a photoperiod of 16:8 (L:D). Larval mortality was recorded daily until 10 days after inoculation. 

### 4.5. Data Analysis

Larval mortality for each HearNPV concentration was corrected based on the control mortality according to the Abbott’s formula [54]. Probit analysis [55] was utilized to determine the median lethal concentration (LC_50_) that caused 50% mortality and the corresponding 95% confidence limit (CL) for each *H. zea* strain. The resistance ratio was calculated by dividing the LC_50_ value of the tested strain by the LC_50_ value of the SS strain. Differences among LC_50_ values for *H. zea* strains were assessed by comparing the 95% confidence interval of the LC_50_ for each strain. Overlapping confidence intervals indicate nonsignificant differences between LC_50_s, whereas non-overlapping intervals indicate significant differences. In addition, the mortality response of *H. zea* to HearNPV was analyzed using a two-way ANOVA with insect strain and HearNPV concentration as the two main factors [55]. The means were separated using the Tukey–Kramer adjustment (α = 0.05). To account for heterogeneity, the mortality of each strain was transformed using arcsine transformation, but the non-transformed data are presented. Furthermore, a correlation analysis was performed utilizing Pearson’s correlation coefficient using GraphPad Prism 9.5.0 [56] to determine the correlation between Bt and HearNPV resistance in *H. zea*. 

## 5. Conclusions

The challenge posed by the evolution of resistance in *H. zea* to various insecticides and Bt proteins makes pest management highly complex. The utilization of biological agents, such as HearNPV, in the management of *H. zea* could potentially play a crucial role in addressing this challenge effectively and sustainably. Understanding the baseline susceptibility of insect pests to insecticides is a crucial component of a proactive resistance monitoring program. In this study, we assessed the response of several field and laboratory strains of *H. zea* to HearNPV and investigated the potential relationship between *Hear*NPV and Bt resistance in *H zea*. We found that *H. zea* strains resistant to Cry Bt proteins demonstrated significantly reduced susceptibility to HearNPV, whereas strains resistant to the Vip3Aa Bt protein exhibited high susceptibility to the virus. Correlation analysis indicated a significant positive relationship between Cry resistance and HearNPV resistance in *H. zea,* whereas no significant correlation was observed between Vip3Aa resistance and HearNPV resistance. Overall, these results suggest that HearNPV has the potential to help manage Vip3Aa resistance in *H. zea*. Therefore, it is beneficial to integrate HearNPV as an alternative management tool in IPM and IRM programs for *H. zea*, given the existing widespread Cry resistance in this insect pest. The adoption of HearNPV for *H. zea* management not only mitigates resistance development in the pest but also offers a reduced-risk insecticide alternative, aligning with the public demand for more sustainable agriculture and a healthier environment. Further investigation is essential to understand the underlying mechanisms responsible for varying levels of susceptibility to HearNPV among the different strains of *H. zea*, as well as the associations between HearNPV susceptibility and Bt resistance. 

## Figures and Tables

**Table 1:** Dose response of laboratory and field-collected *H. zea* strains against HearNPV.

Insect Strain	Host	n ^a^	LC_50_ (95% FL) (OBs/mL)	Slope ± SE	*x^2^*	df	Resistance Ratio (RR) ^b^
2021							
SS	Lab diet	840	6.5 × 10^3^ (4.9 × 10^3^, 8.4 × 10^3^)	1.7 ± 0.2	119	22	1
CRY-RR	Lab diet	840	71.0 × 10^3^ (54.6 × 10^3^, 99.8 × 10^3^)	1.4 ± 0.1	104	28	10.9 *
VIP-RR-70	Lab diet	1050	10.5 × 10^3^ (8.0 × 10^3^, 14.0 × 10^3^)	1.3 ± 0.1	128	28	1.6
Thrall-TX	Intrasect corn	560	147.3 × 10^3^ (64.4 × 10^3^, 3625.0 × 10^3^)	1.3 ± 0.4	9.5	22	22.7 *
Malone-TX	DoublePro corn	616	62.6 × 10^3^ (43.1 × 10^3^, 100.0 × 10^3^)	1.2 ± 0.2	60.4	22	9.5 *
Winnsboro-LA	DoublePro corn	630	66.9 × 10^3^ (33.3 × 10^3^, 241.3 × 10^3^)	0.8 ± 0.2	25.2	22	10.3 *
Alexandria-LA	Trecepta corn	336	134.4 × 10^3^ (54.2 × 10^3^, 1623.9 × 10^3^)	0.9 ± 0.2	14.7	16	20.7 *
2022							
SS	Lab diet	448	2.8 × 10^3^ (2.1 × 10^3^, 3.7 × 10^3^)	1.9 ± 0.2	86.6	22	1
VIP-RR-15	Lab diet	448	1.8 × 10^3^ (0.8 × 10^3^, 3.1 × 10^3^)	1.0 ± 0.2	39.2	22	0.7
TRE-RR	Lab diet	238	1.6 × 10^3^ (0.9 × 10^3^, 2.4 × 10^3^)	1.2 ± 0.2	47.7	16	0.6
Epps-LA	Crimson clover	336	490.0 × 10^3^ (100.0 × 10^3^, 4.9 × 10^8^)	0.6 ± 0.2	10.2	16	175.0 *
Leland-MS	NBT corn	448	5.5 × 10^3^ (4.0 × 10^3^, 7.5 × 10^3^)	1.7 ± 0.2	89.6	22	2.0 *
Marianna-AR	NBT corn	448	5.0 × 10^3^ (4.0 × 10^3^, 6.3 × 10^3^)	1.8 ± 0.1	147	22	1.8 *
Taylor-TX	NBT corn	448	10.2 × 10^3^ (6.2 × 10^3^, 17.0 × 10^3^)	0.6 ± 0.1	45.4	22	3.6 *

^a^ Total number of larvae assayed. ^b^ Resistance ratios were calculated by dividing the LC_50_ value of the test population by the LC_50_ of the SS strain. * Indicates significant resistance ratios based on non-overlapping 95% CIs.

**Table 2:** Bt resistance ratio of field-collected and laboratory strains of *H. zea*.

Insect Strain	Cry1Ac	Cry2Ab2	Vip3Aa39	Reference
CRY-RR	779.3 *	387.8 *	0.4	[32]
VIP-RR-70	6.3	1.9	>892.8 *	[33]
VIP-RR-15	2.0	1.2	>892.8 *	[33]
TRE-RR	264.8 *	495.3 *	>95.7 *	[33]
Thrall-TX	2,787,333 *	64.8 *	<0.09	[34]
Malone-TX	539.6 *	67.3 *	<0.09	[34]
Taylor-TX	55,028.6 *	12.0 *	0.2	[35]
Winnsboro-LA	154.6 *	79.4 *	0.21	[34]
Alexandria-LA	/	5.31	<0.09	[34]
Epps-LA	351.1 *	740.7 *	1.9	[35]
Leland-MS	72,150.0 *	13.2 *	0.04	[35]
Marianna-AR	1498.6 *	8.0	0.3	[35]

* Indicates significant resistance ratios (≥10-fold).

## Data Availability

The raw data supporting the conclusions of this article will be made available by the authors on request.
